# Needle Arthroscopic All-Inside Repair of Meniscal Tears Under Local Anesthesia

**DOI:** 10.1016/j.eats.2021.05.020

**Published:** 2021-08-28

**Authors:** Tobias Stornebrink, Robbert A.H.E. van Dijck, Dirk Douven, Gino M.M.J. Kerkhoffs

**Affiliations:** a Amsterdam UMC, University of Amsterdam, Department of Orthopedic Surgery, Amsterdam Movement Sciences, Amsterdam, The Netherlands; b Academic Center for Evidence-based Sports Medicine (ACES), Amsterdam, The Netherlands; c Amsterdam Collaboration for Health & Safety in Sports (ACHSS), International Olympic Committee (IOC) Research Center Amsterdam UMC, Amsterdam, The Netherlands; d Bergman Clinics, Breda, The Netherlands

## Abstract

Needle arthroscopy has experienced a substantial increase in image quality due to technical innovation, which has brought needle arthroscopic interventional possibilities along. Repair of meniscal tears is gaining popularity relative to meniscectomy and may be such a procedure that is suitable for needle arthroscopy. We here present a needle arthroscopic technique for all-inside repair of meniscal tears in the red zone and red–white zone. With the use of local anesthesia only, the procedure is easy to perform for the surgeon and well-tolerable for the patient. Compared with conventional approaches to meniscal repair, needle arthroscopy may result in improved patient experience, decreased soft-tissue trauma, speedier recovery, and less need for personnel and hospital facilities. Combined, the advantages may lead to decreased overall costs as well.

A meniscal tear is a common problem. In the United States alone, approximately 1 million meniscal surgeries are performed each year.^1^ This rather large demand necessitates an effort to innovate and try to provide increased quality of care at lower costs. Indeed, surgical treatment of meniscal injuries has evolved from open resection in the past to arthroscopic repair in the present.^2^ Needle arthroscopy now offers adequate image quality^3^ and provides the opportunity to further innovate the delivery of care. We here describe a technique for needle arthroscopic, all-inside repair of meniscal tears in the red zone and red–white zone, as it is performed by one of the senior authors (R.v.D.). This approach can be performed under local anesthesia and in the procedure room. The study was conducted in agreement with the 1964 Helsinki Declaration and its later amendments and was approved by the Medical Ethics Committee of the University of Amsterdam (AMC), with reference number W20_357 # 20.412.

## Surgical Technique (With Video Illustration)

Video 1 presents the technique of the entire procedure step by step, from anesthesia to closure.

### Anesthesia

**Fig 1:**
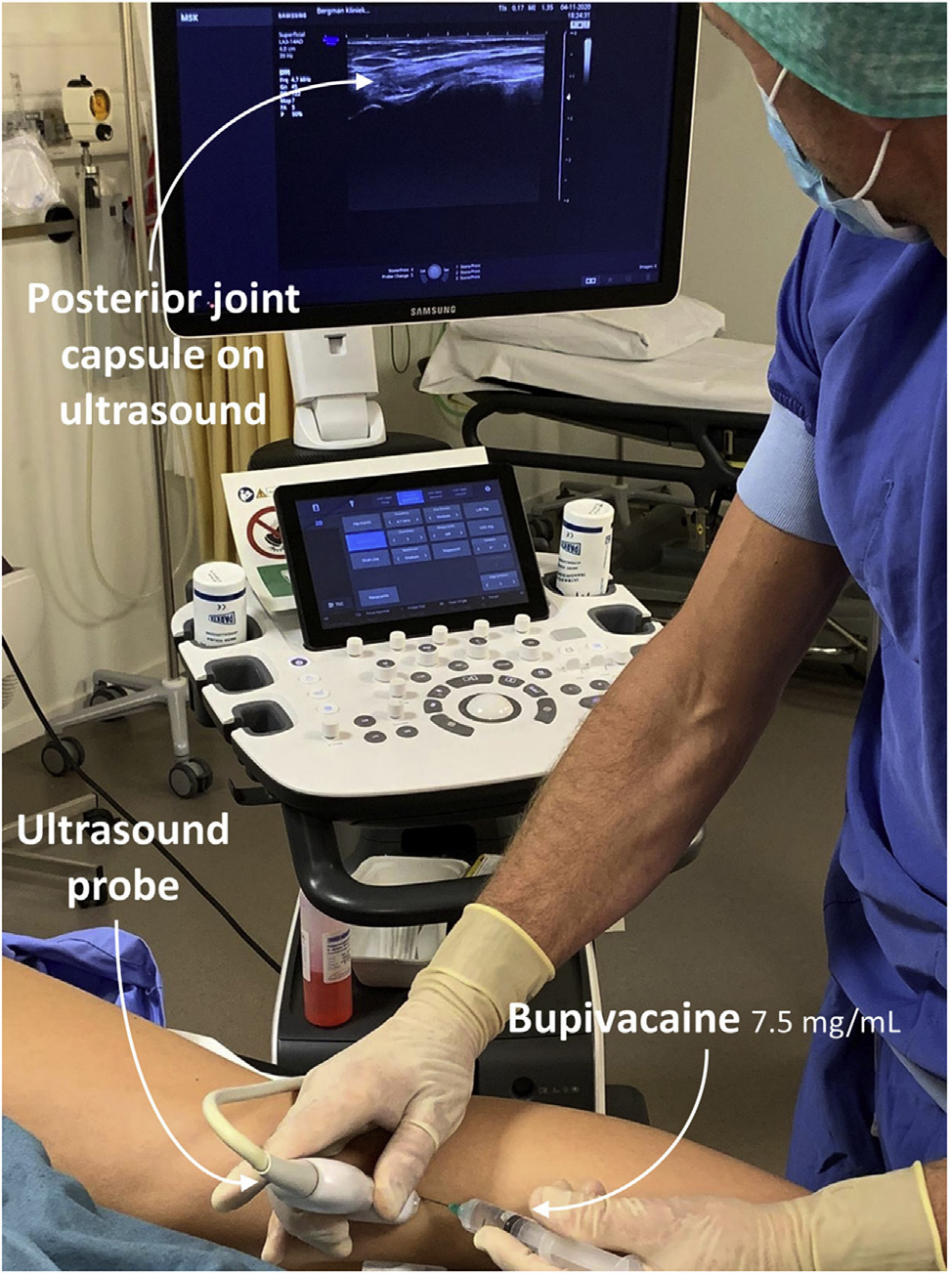
The right knee seen from a posteromedial angle. The posterior joint capsule is anesthetized with bupivacaine 7.5 mg/mL. Ultrasonography is used for guidance. Anesthesia is applied in the holding room before surgery. This ensures that the anesthetic has taken sufficient effect for a painless procedure and expedites operating room procedures. Special attention is paid to careful infiltration of the joint capsule, as this is well-innervated tissue.

Local anesthesia is applied in the holding room (Fig 1). Standard anteromedial and anterolateral portals are identified by palpation. In sterile fashion, 10 cc of levobupivacaine 7.5 mg/mL is injected along each portal tract—from skin to joint capsule—and intra-articular. Close attention should be paid to proper anesthesia of the joint capsule, as this is well innervated tissue. In addition, the posterior joint capsule and the medial collateral ligament are anesthetized. Ultrasonography is used to guide these latter 2 injections.

### Patient Setup

**Fig 2:**
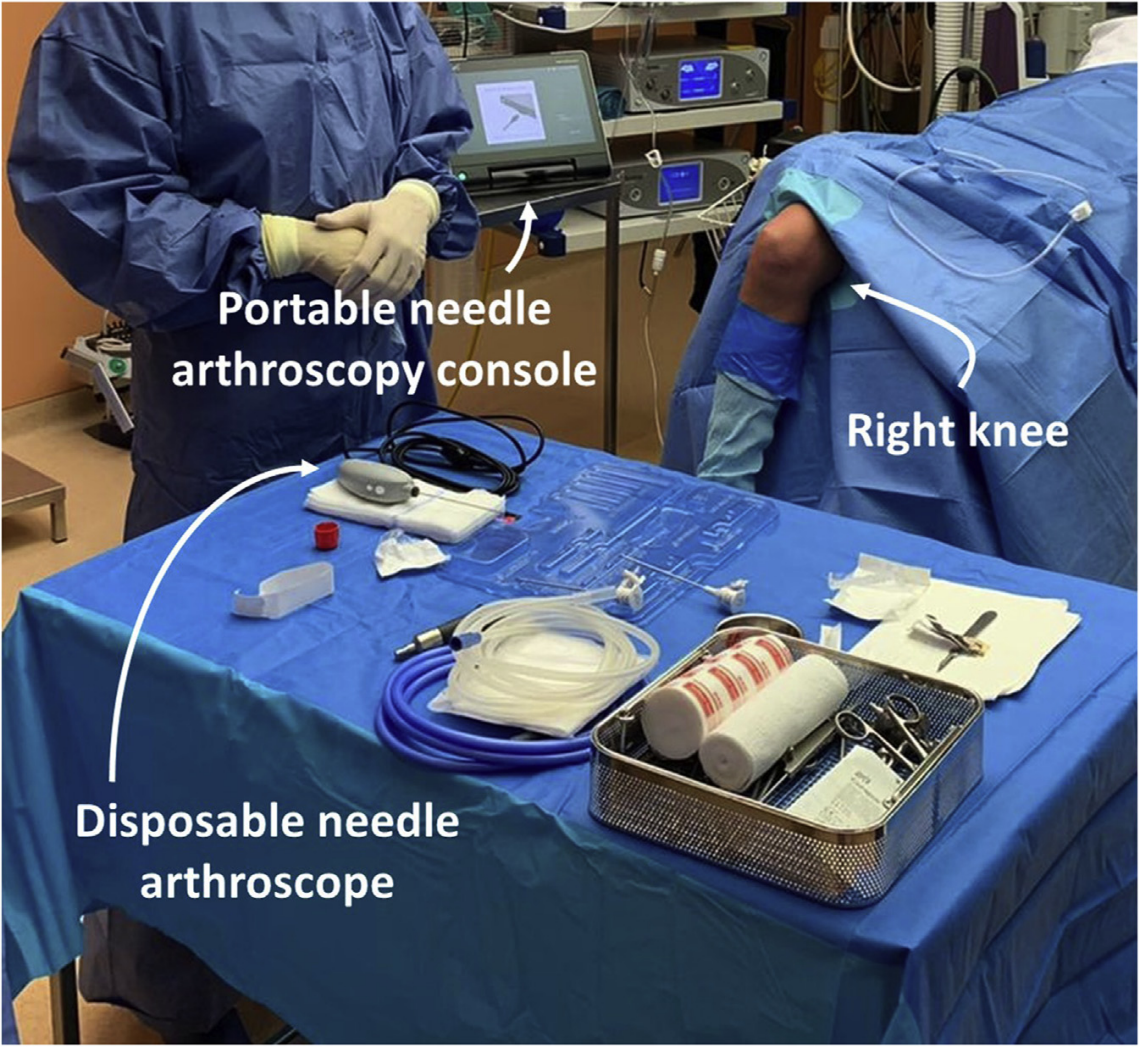
The operative setting just before the start of the procedure, with the right knee seen from an anteromedial perspective. The procedure is performed in a room with operating theater–quality air control, but without an anesthesia team. Arthroscopic imaging is processed by a portable, tablet-like console and can be routed to overhead monitors if preferred.

The patient is moved to the operating theater or procedure room. Be sure to adhere to local directives regarding control of air quality. The patient is positioned in supine position. Leg rests are removed to allow for 90° knee flexion. A tourniquet is applied at the thigh and inflated to 250 mm Hg. The surgical field is disinfected and covered with sterile draping (Fig 2).

### Arthroscope Introduction

With the knee in 90° flexion, a 2.2-mm diameter cannula is loaded with a sharp obturator. This cannula is then percutaneously inserted in the joint space through a standard lateral portal—no incisions are needed. The obturator is removed and the 1.9-mm diameter needle arthroscope (NanoScope; Arthrex, Naples, FL) is inserted through the cannula. This needle arthroscope is semi-rigid and has a 0° direction of view. Either a syringe or an arthroscopic pump is connected to the cannula and the joint is distended with sterile saline. The anterolateral viewing portal is hence simultaneously used as inflow portal.

**Fig 3:**
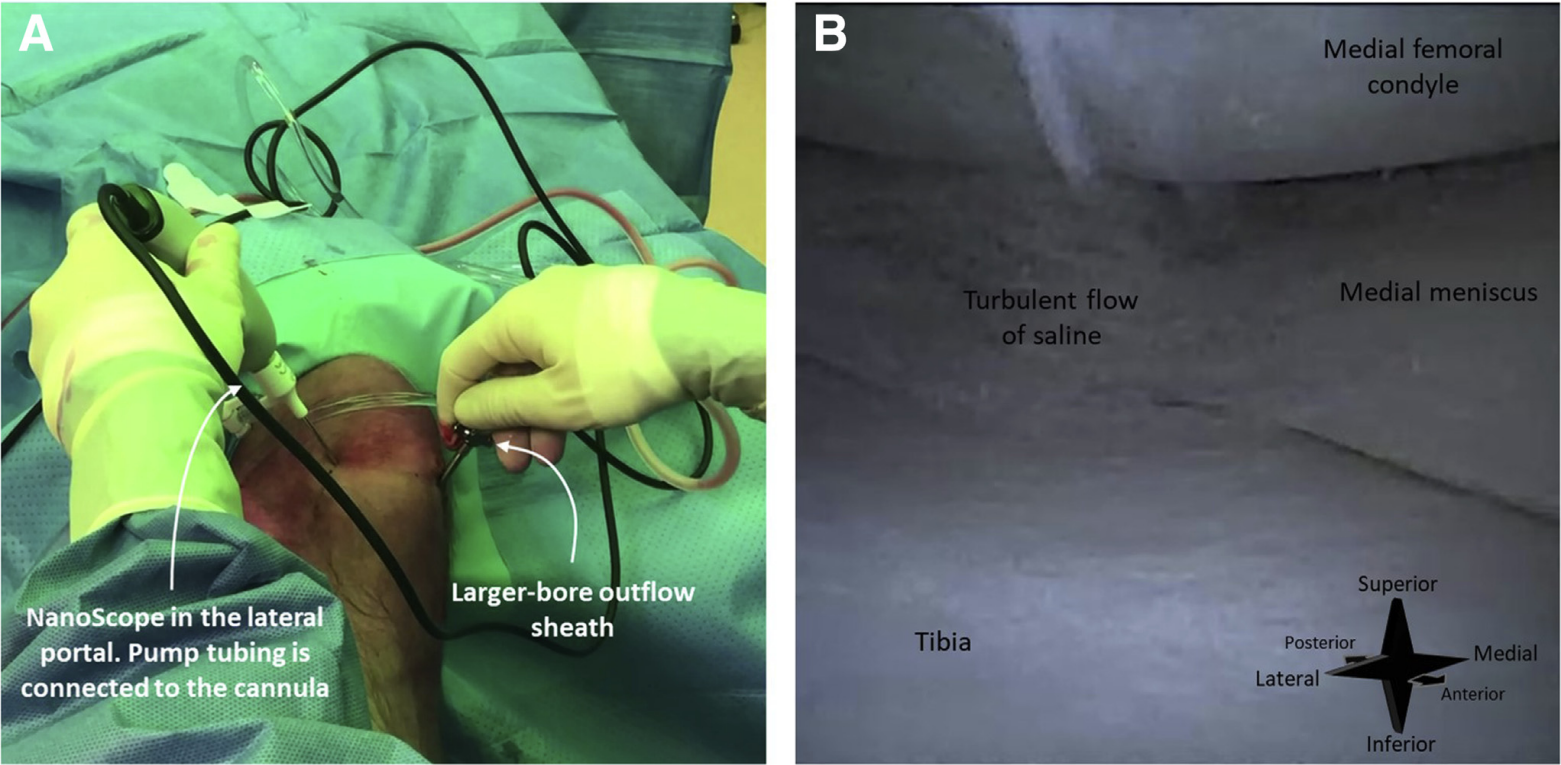
A right knee seen from anterior (A). The needle arthroscope is inserted intra-articular through an anterolateral portal. The corresponding arthroscopic view is shown in image (B). An outflow sheath is connected to a suctioning device and inserted intra-articular through the anteromedial portal. This results in increased flow of saline (B) and flushes the joint, clearing any blood or debris.

Due to its small-bore diameter, inflow of saline through the needle arthroscopic cannula is lower compared with traditional arthroscopy. This may result in obscured vision in the case of extensive intra-articular blood or debris. We therefore recommend keeping a separate, large-bore outflow sheath connected to a suctioning device ready for use. This separate outflow sheath can be temporarily inserted through the working portal. This establishes increased flow and flushes the joint (Fig 3).

**Fig 4:**
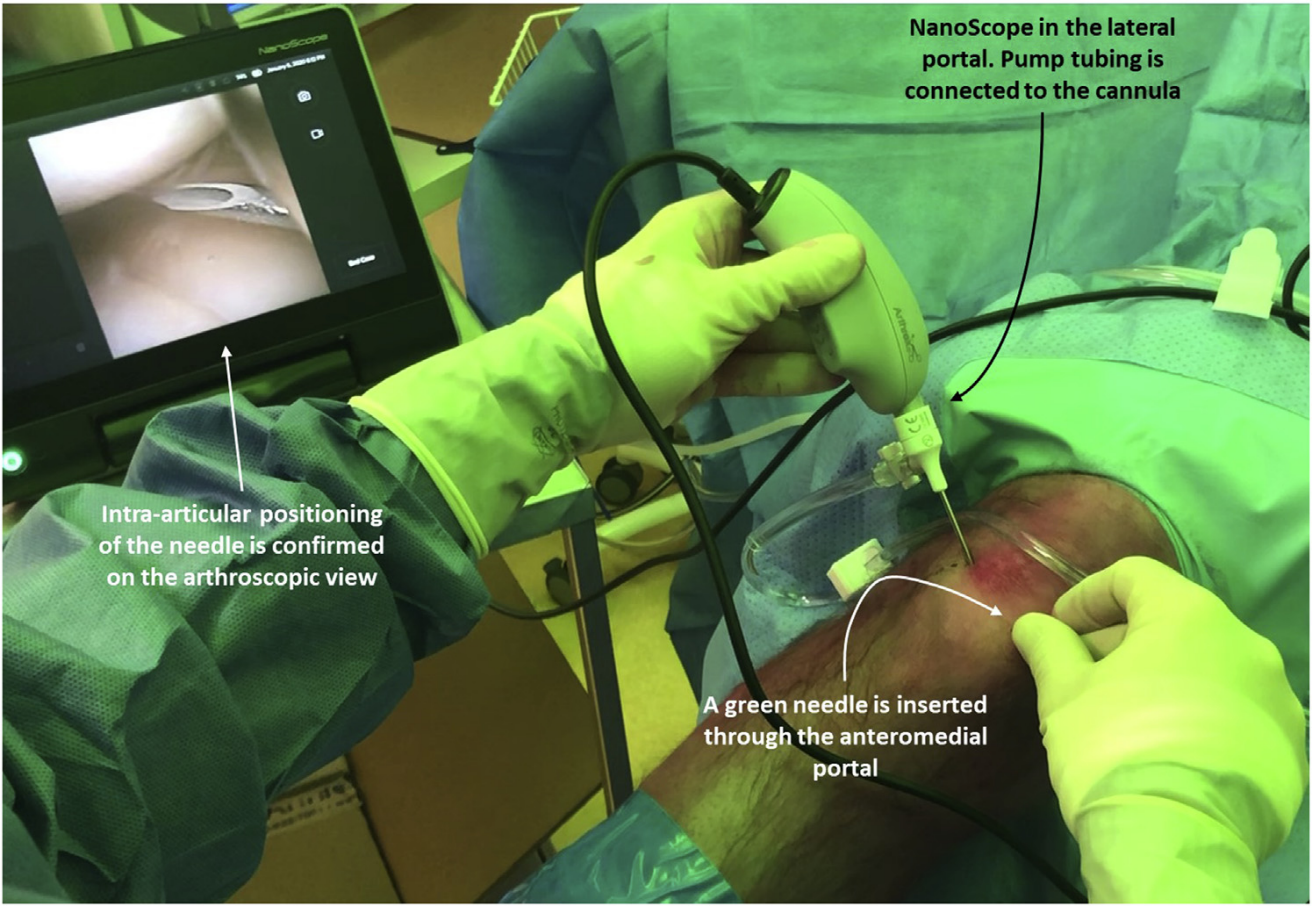
The right knee as seen from an anteromedial perspective. The needle arthroscope is inserted through the anterolateral portal. A green needle is used to locate the anteromedial portal. It is seen intra-articular on the needle arthroscopic imaging console, which confirms correct placement of the portal.

A standard diagnostic arthroscopy is performed. The anteromedial portal is used as the working portal. It is created under arthroscopic visualization (Fig 4) with a 3-mm stab incision of the skin and blunt penetration of the joint capsule with a mosquito clamp. Instruments such as a probe and the suturing device can then be inserted through this second portal percutaneously, without a cannula.

### Meniscus Repair

**Fig 5:**
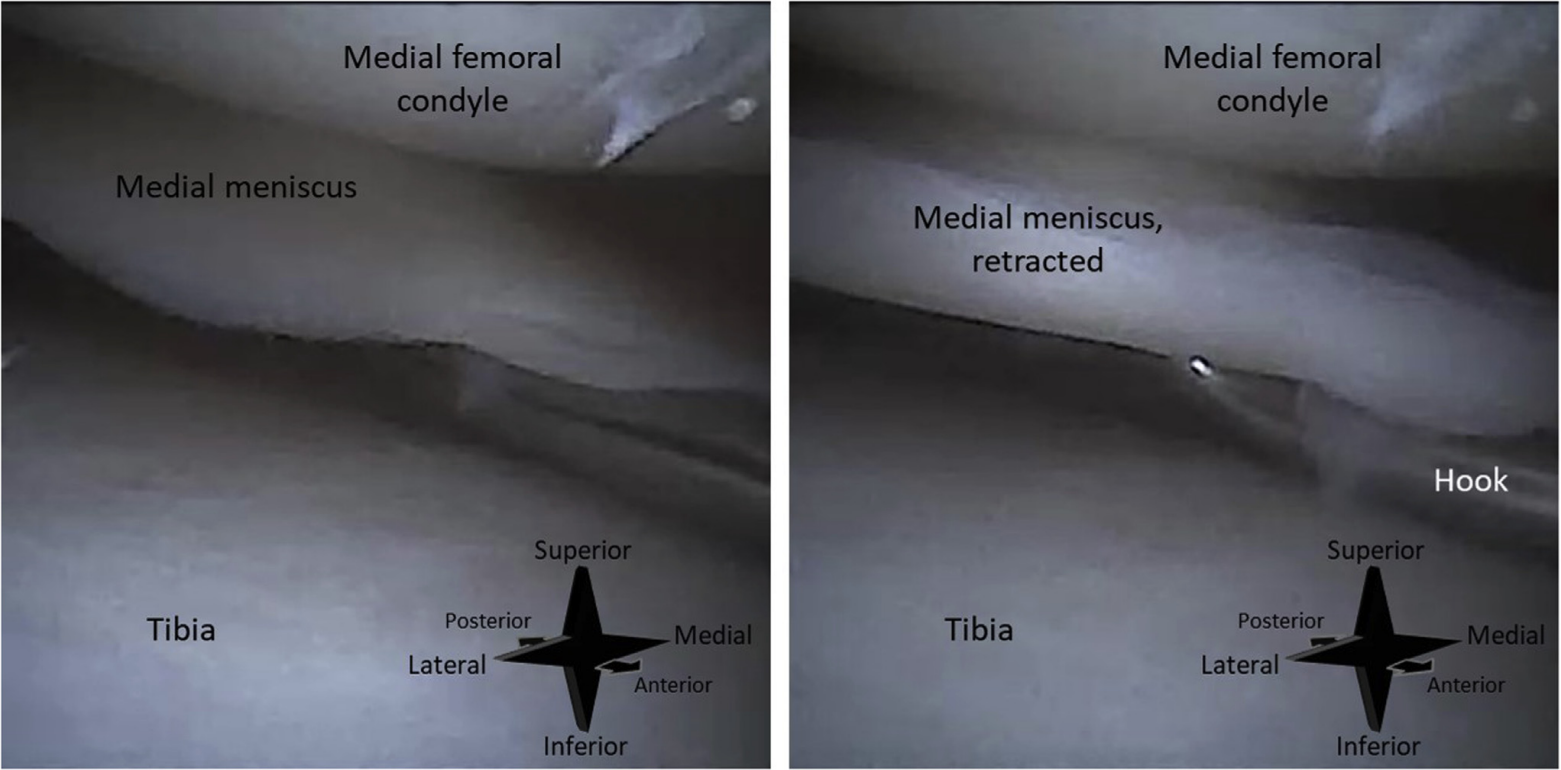
Needle arthroscopic imaging of a right knee, as obtained with the camera inserted through an anterolateral portal. The meniscal tear is probed with a hook probe inserted through an anteromedial portal. In image (A), no tension is applied. In image (B), the tear is hooked and retracted, showing instability of the meniscus.

**Fig 6:**
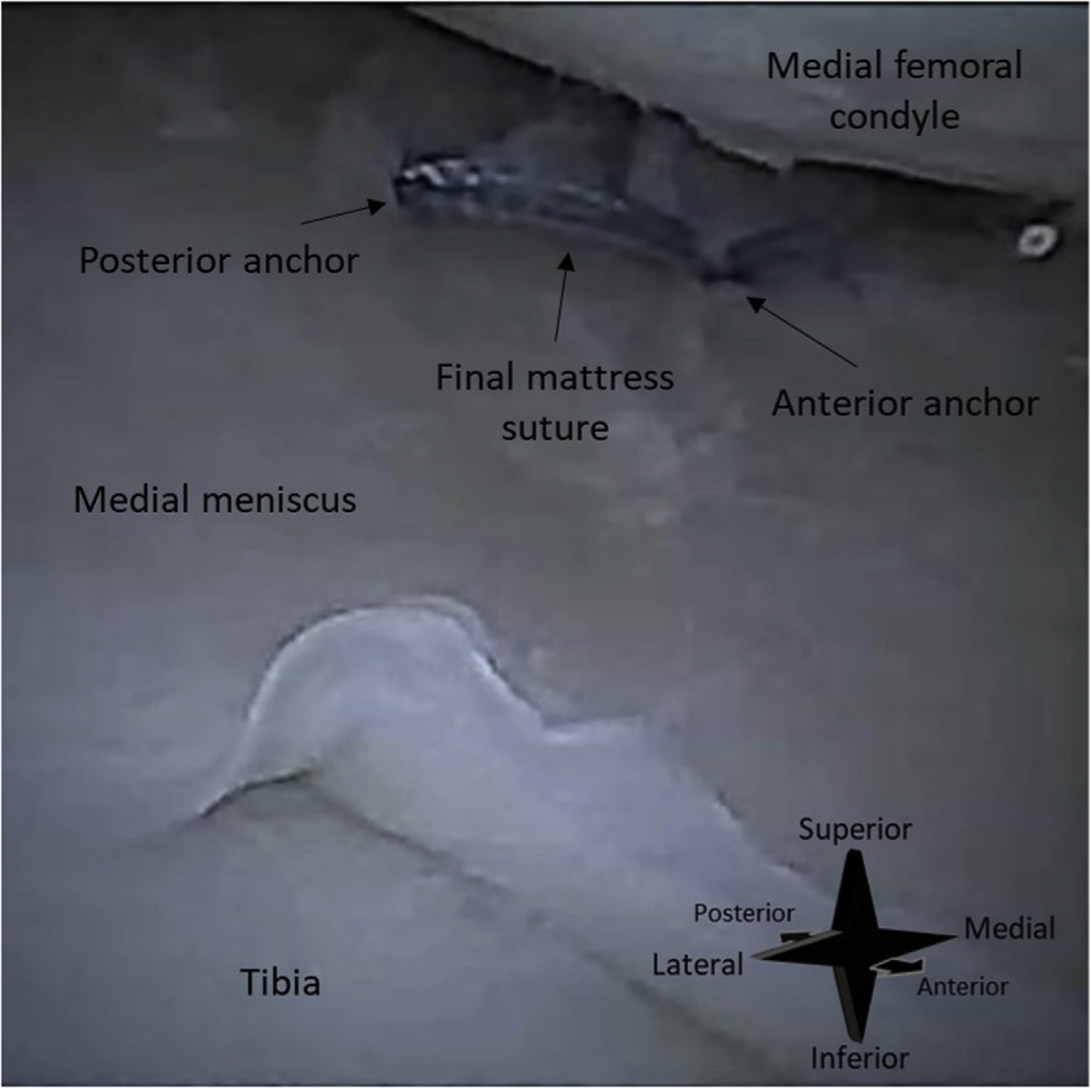
Needle arthroscopic image of a right knee, as obtained with the camera inserted through an anterolateral portal. The final result that the procedure aims for is shown. A mattress suture was implanted for a vertical tear of the medial meniscus. The base of the 2 anchors of the mattress suture is not visible, as they are inserted through the entire meniscus and joint capsule.

**Fig 7:**
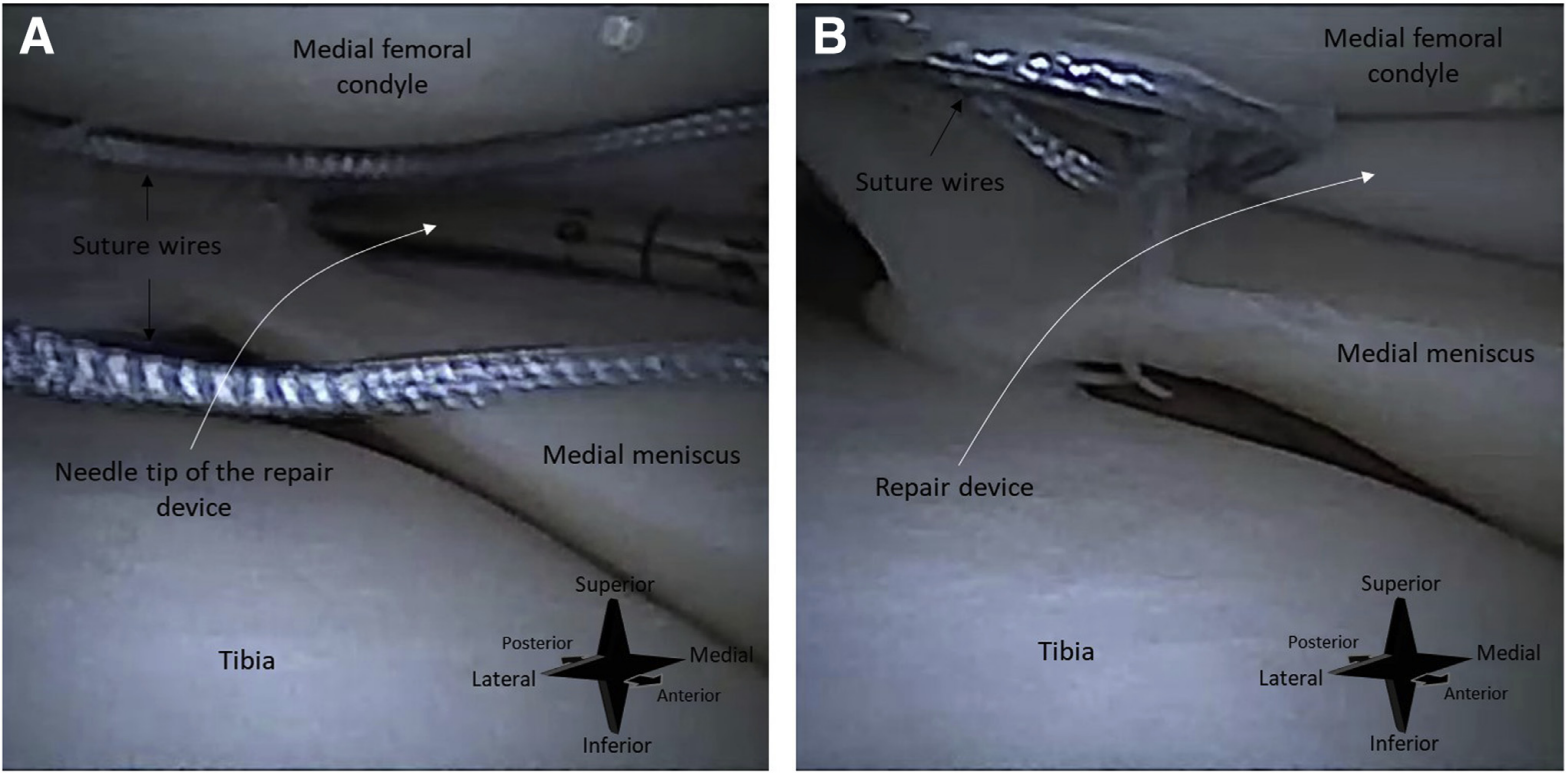
Needle arthroscopic imaging of a right knee, as obtained with the camera inserted through an anterolateral portal. The all-inside repair device is inserted through an anteromedial portal. In image (A), the needle tip of the repair device is positioned on the medial meniscus. In image (B), the repair device had been advanced through the meniscus and joint capsule and suture anchor can be deployed.

An all-inside suturing device (FiberStitch; Arthrex) is used for the repair. First, the meniscal tear is identified with help of a hooked probe (Fig 5). The depth of the meniscus is measured with a probe with graduation lines. The depth stop of the suturing device is set at a length 2 mm longer than the meniscal depth. This ensures that the device deploys its implant through the entire meniscus. The goal of the suturing device is to deliver a mattress suture with 2 anchors that are deployed behind the joint capsule (arthroscopic view of a final mattress suture in Fig 6). For each anchor, the sharp tip of the device is advanced through the meniscus and joint capsule (Fig 7). The anchors are then deployed by rolling the deployment wheel backward and subsequently forward until a hard stop.

**Fig 8:**
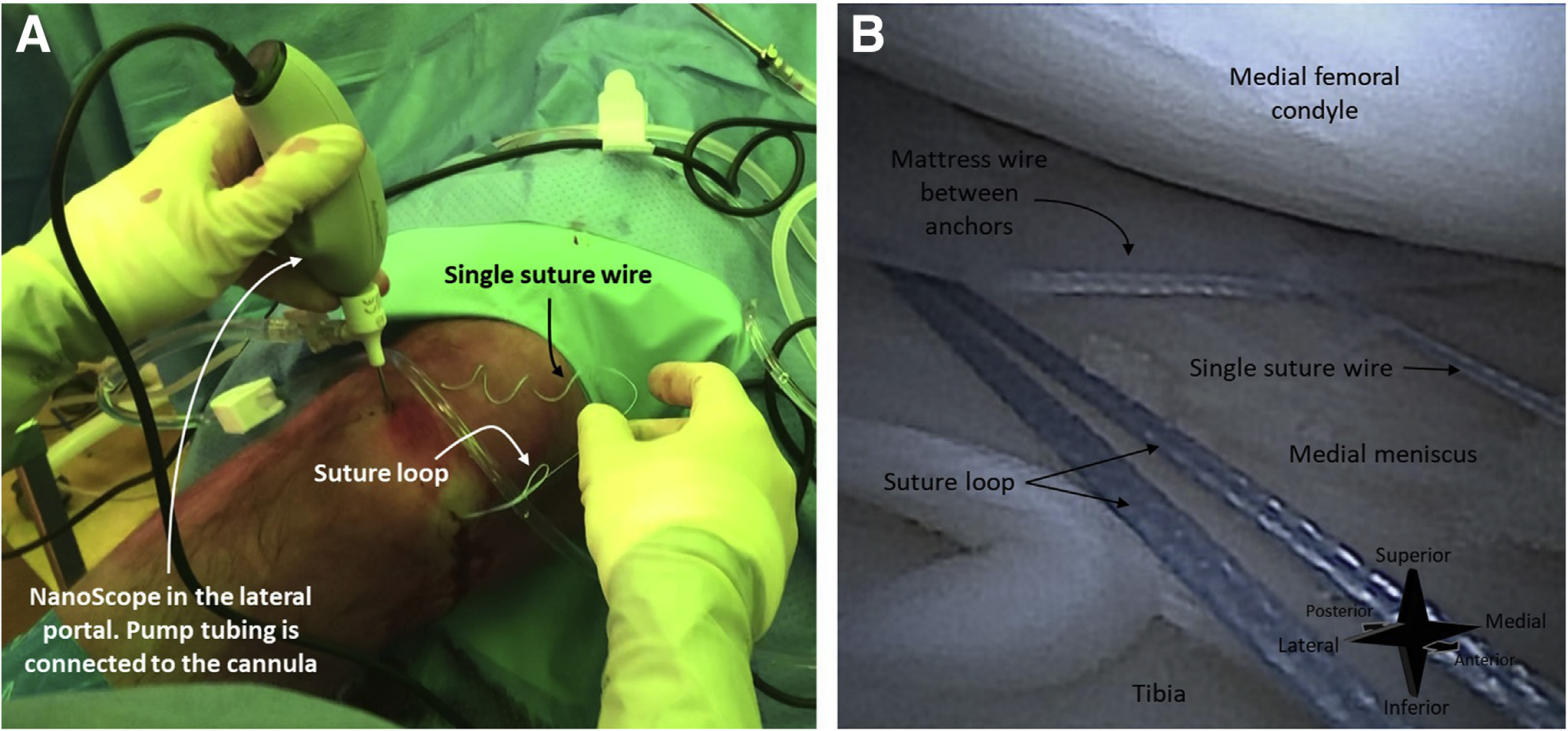
A right knee seen from an anteromedial perspective (A). The needle arthroscope is inserted intra-articular through an anterolateral portal. The corresponding arthroscopic view is shown in image (B). Once both suture anchors are deployed, 3 parts of the suture system can be distinguished: (1) a first mattress wire between the anchors, (2) a single wire that originates from the first anchor, and (3) a loop that originates from the second anchor.

**Fig 9:**
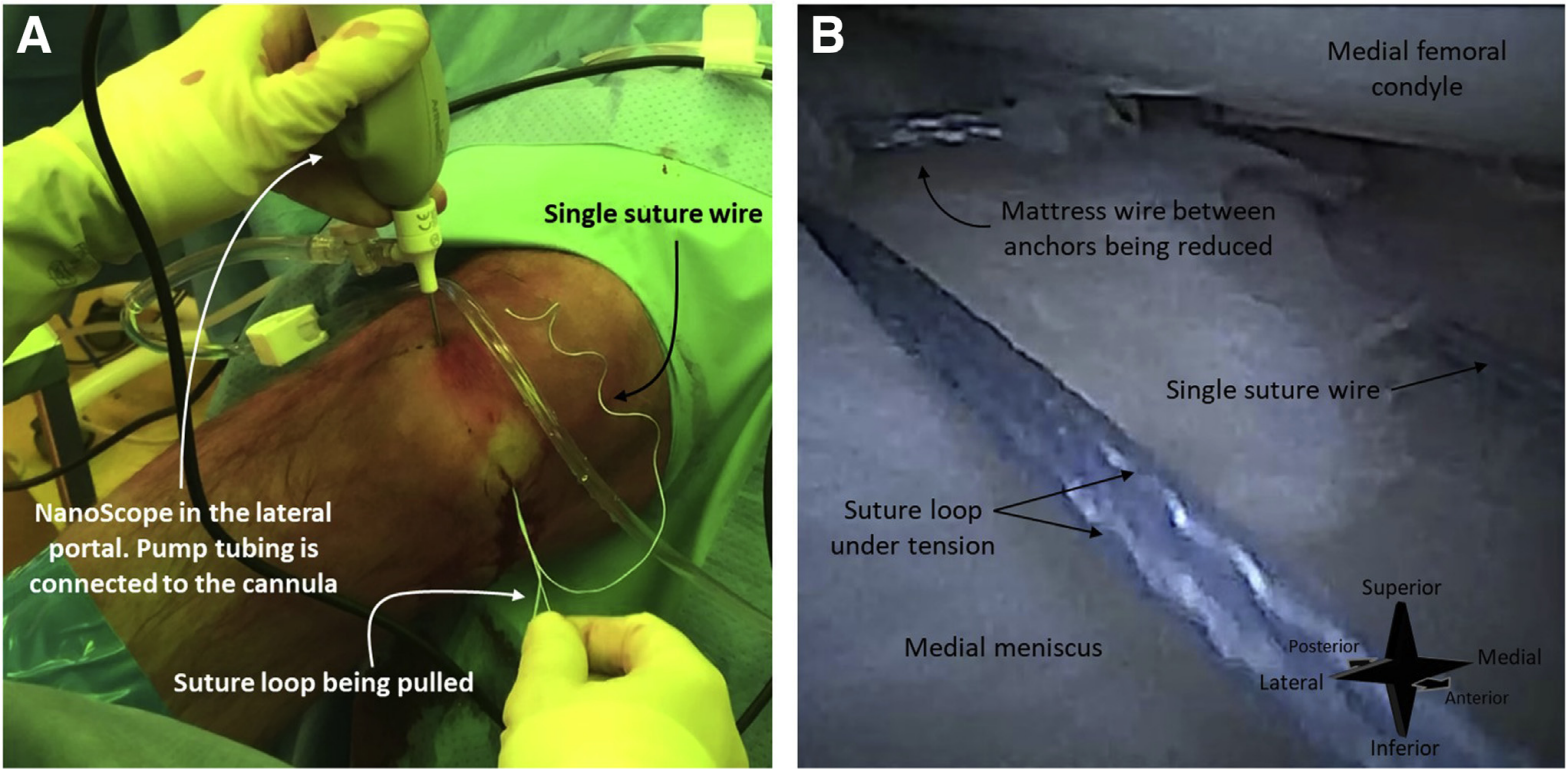
A right knee seen from an anteromedial perspective (A). The needle arthroscope is inserted intra-articular through an anterolateral portal. The corresponding arthroscopic view is shown in image (B). By pulling the suture loop, the mattress wire between the anchors is tightened, and the meniscal tear is reduced.

**Fig 10:**
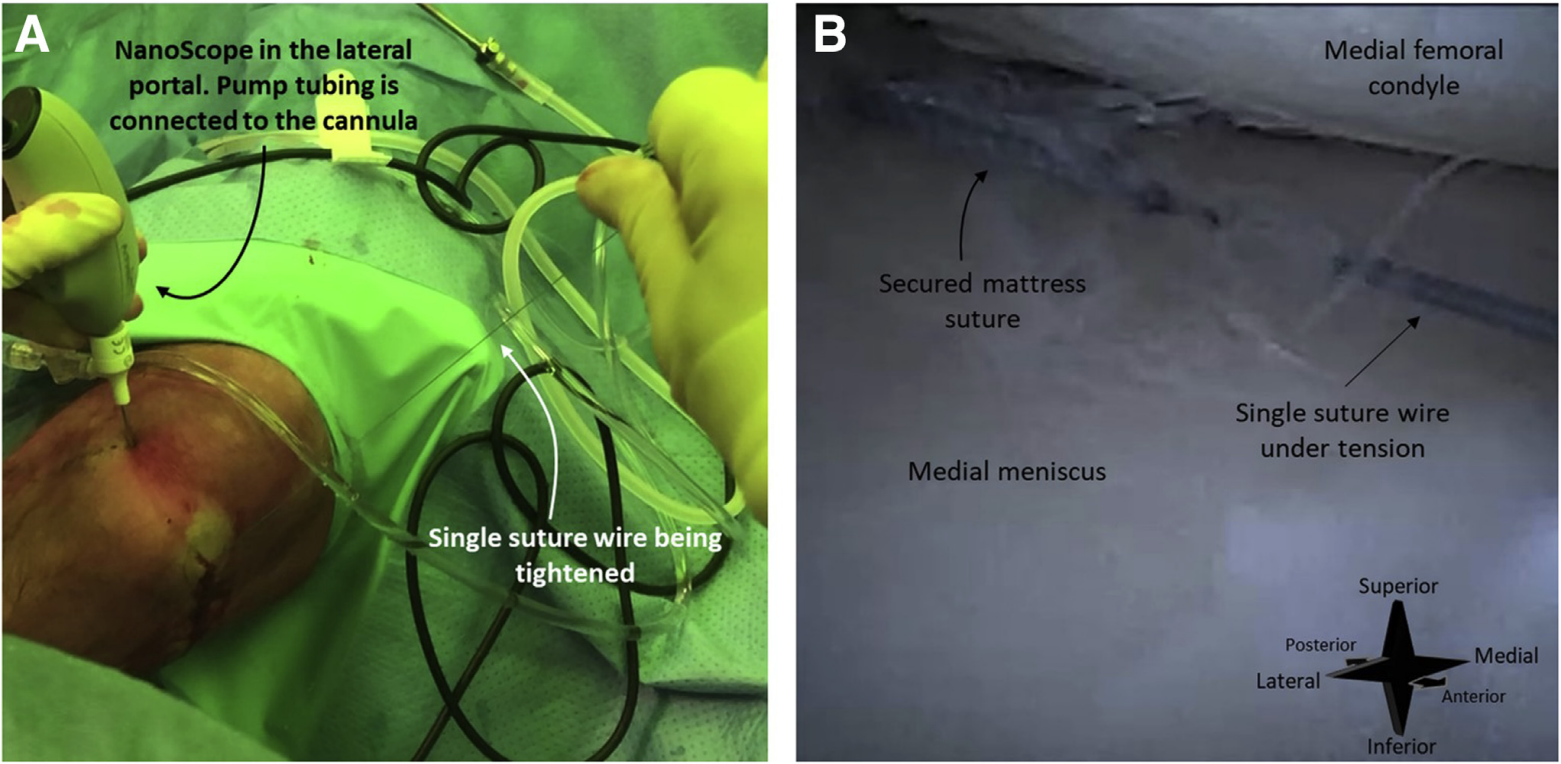
A right knee seen from an anteromedial perspective (A). The needle arthroscope is inserted intra-articular through an anterolateral portal. The corresponding arthroscopic view is shown in image (B) Once the meniscal is sufficiently reduced, the mattress suture is secured by pulling the single-suture wire. This tightens the loop.

Once both anchors are deployed and the device is removed from the joint, a mattress wire between the anchors, a suture loop, and a single suture wire will be visible (Fig 8). By pulling the loop, the mattress suture wire between the anchors is tightened and the meniscal tear is reduced (Fig 9). Upon sufficient reduction of the tear, the suture loop is tightened to reduce the suture loop. This secures the entire mattress implant (Fig 10).

The single-suture wire is now loaded on the knot pusher. The knot pusher is advanced intra-articular and down to its anchor, whilst maintaining tension on the single-suture wire. The knot pusher is positioned right on the mattress knot, and the single suture wire is cut. Finally, A 2-mm or 3-mm diameter shaver is used to address meniscal and chondral fraying if present.

### Closure

**Fig 11:**
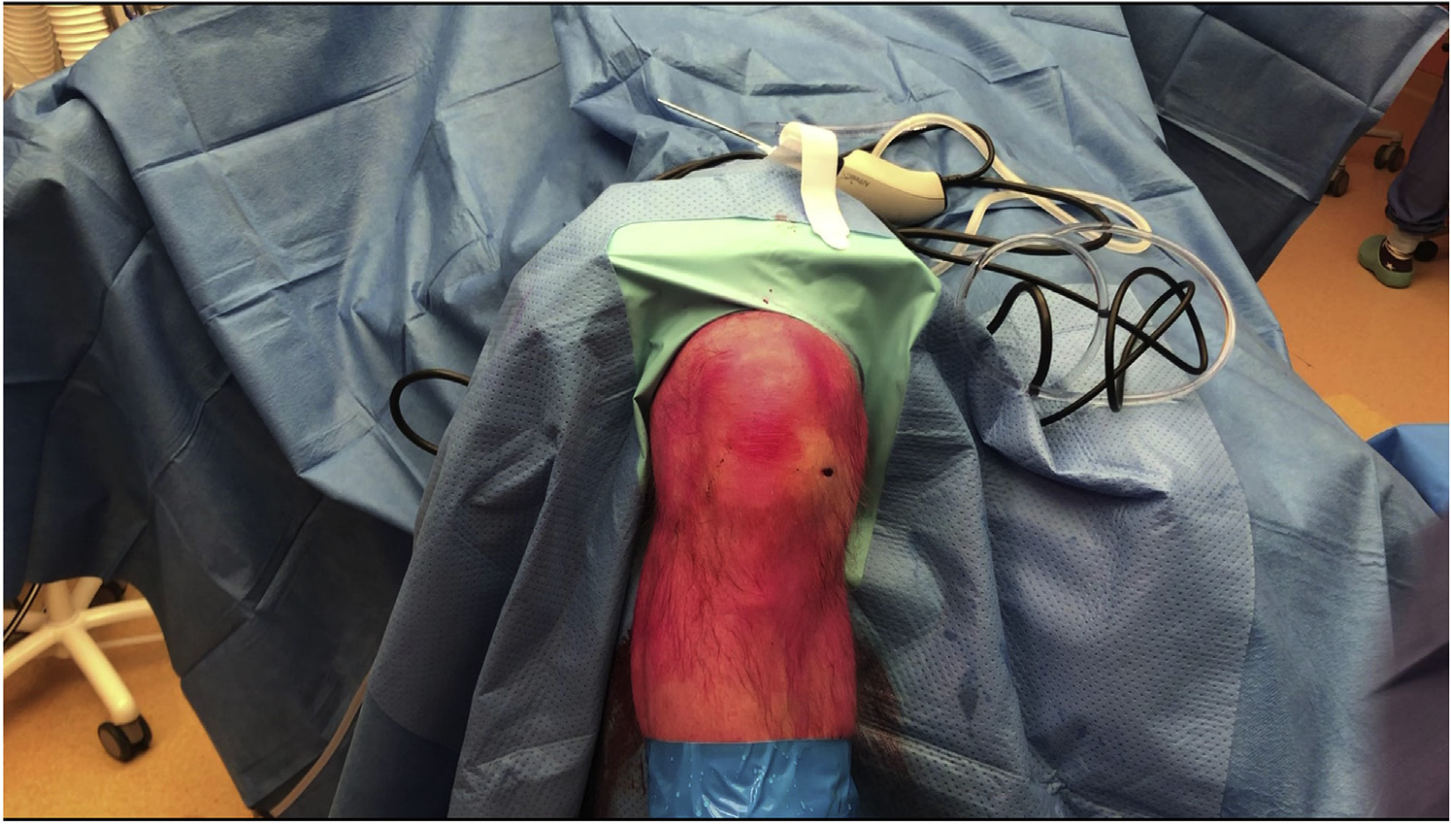
The right knee as seen from an anterior perspective. The camera portal can usually be closed with sterile wound closure strips or a simple band aid. The working portal is closed with a simple wire suture.

The joint is flushed with sterile saline and subsequently aspirated to dryness through the cannula. All instruments are removed. Sterile wound closure strips or a simple band aid are usually sufficient for skin closure of the percutaneous camera portal (Fig 11). A simple wire suture is used for the working portal. Standard postoperative rehabilitation is started.

## Discussion

The needle arthroscopic technique presented here provides a minimally invasive approach to repair of meniscal tears in the red zone or red–white zone. It is easy to perform for the surgeon and well-tolerated by the patient under local anesthesia. Needle arthroscopy was first introduced in the 1990s. Inferior image quality hampered its adoption and excluded any interventional indications.^4^ Recent innovation however markedly increased image quality and introduced needle arthroscopic instruments of a similarly small diameter.^5^ This innovation has fueled the use of needle arthroscopy, with diagnostic and interventional indications arising in the ankle, knee, and shoulder.^6^, ^7^, ^8^

Needle arthroscopy seems to be perfectly suited for repair of isolated meniscal tears. To the patient, a meniscal suture may sound like a relatively simple operation. Yet, conventional knee arthroscopy still constitutes an invasive procedure that causes significant soft-tissue trauma. In the vast majority of cases, it necessitates aggressive forms of anesthesia, with their own risk of complications. Various recent needle arthroscopic techniques have emerged that are successfully performed under local anesthesia,^9^, ^10^, ^11^ and we expect the advantages of needle arthroscopic repair of meniscal tears (Table 1) to include an improved patient experience, decreased soft-tissue trauma, speedier recovery and less need for personnel and hospital facilities—a feature that proves valuable in current times of scarcity. Altogether, needle arthroscopy may result in a substantial cost-reduction as well. We do recommend to pay attention to potential downsides when considering needle arthroscopy for a patient (Table 2).

**Table 1:** Advantages of Needle Arthroscopic Meniscal Repair Compared With Traditional Arthroscopy

Improved patient experience
Decreased soft-tissue trauma
Speedier recovery
Less need for personnel and hospital facilities if performed under local anesthesia
Potential for cost-savings

**Table 2:** Pitfalls of Needle Arthroscopic Meniscal Repair Compared With Traditional Arthroscopy

The 0° viewing angle requsires requires a learning curve
The small instruments may increase difficulty and procedure time in case of large tears or extensive concomitant injury
If performed under local anesthesia, unexpected discovery of certain concomitant injuries may require conversion to general anesthesia or a second procedure at a later stage
If performed under local anesthesia, one should pay close attention to anesthesia of the joint capsule and medial (for medial tears) or lateral (for lateral tears) collateral ligaments. Improper anesthesia of these tissues will result in a painful procedure.
Due to its small-bore diameter, inflow of saline through the needle arthroscopic cannula is lower compared with traditional arthroscopy. This may result in obscured vision in case of extensive intra-articular blood or debris. We recommend to keep a separate, large-bore outflow sheath connected to a suctioning device ready for use. This separate outflow sheath can be temporarily inserted through the working portal to increase flow of saline and flush the joint.
Arthroscope and instrumentation are less rigid than traditional arthroscopic equipment, which requires a learning curve.

In conclusion, needle arthroscopy seems to be the next innovation that can increase the quality of meniscal repair, whilst simultaneously lowering its costs and we have started a prospective, comparative evaluation of these alleged merits.

## Supplementary Data

Video 1 A step-by-step approach to needle arthroscopic, all-inside repair of meniscal tears under local anesthesia is shown—from anesthesia to the patient leaving the operating room independently. We use the procedure for tears in the red and red–white zones. The depicted case concerns a patient with a traumatic tear in the medial meniscus of the right knee. Throughout the video, the anteromedial portal is used as viewing portal and the anterolateral portal is used as working portal.

ICMJE author disclosure forms
